# J-domain proteins promote client relay from Hsp70 during tail-anchored membrane protein targeting

**DOI:** 10.1016/j.jbc.2021.100546

**Published:** 2021-03-17

**Authors:** Hyunju Cho, Woo Jun Shim, Yumeng Liu, Shu-ou Shan

**Affiliations:** Division of Chemistry and Chemical Engineering, California Institute of Technology, Pasadena, California, USA

**Keywords:** molecular chaperone, Hsp70, Hsp40, tail-anchored protein, membrane proteins, protein targeting, CTD, C-terminal domain, EV, empty vector, GET, guided entry of tail-anchored protein, JDP, J-domain protein, NBD, nucleotide binding domain, SND, SRP-independent, SBD, substrate binding domain, SRP, signal recognition particle, TMD, transmembrane domain, TPR, tetratricopeptide repeat

## Abstract

J-domain proteins (JDPs) play essential roles in Hsp70 function by assisting Hsp70 in client trapping and regulating the Hsp70 ATPase cycle. Here, we report that JDPs can further enhance the targeting competence of Hsp70-bound client proteins during tail-anchored protein (TA) biogenesis. In the guided-entry-of-tail–anchored protein pathway in yeast, nascent TAs are captured by cytosolic Hsp70 and sequentially relayed to downstream chaperones, Sgt2 and Get3, for delivery to the ER. We found that two JDPs, Ydj1 and Sis1, function in parallel to support TA targeting to the ER *in vivo*. Biochemical analyses showed that, while Ydj1 and Sis1 differ in their ability to assist Hsp70 in TA trapping, both JDPs enhance the transfer of Hsp70-bound TAs to Sgt2. The ability of the JDPs to regulate the ATPase cycle of Hsp70 is essential for enhancing the transfer competence of Hsp70-bound TAs *in vitro* and for supporting TA insertion *in vivo*. These results demonstrate a role of JDPs in regulating the conformation of Hsp70-bound clients during membrane protein biogenesis.

The Hsp70 family comprises a central hub of the chaperone network that maintains cellular protein homeostasis and functions in every stage of the protein life cycle, from *de novo* folding, protein transport, to aggregate remodeling and degradation (1, 2, 3). The function of Hsp70s is governed by the ATPase cycle in its nucleotide binding domain (NBD), which is tightly coupled to interactions with cochaperones. In the ATP-bound state, the α-helical lid of the Hsp70 substrate binding domain (SBD) docks against the NBD and is away from the peptide binding site in the SBD, allowing rapid client binding and dissociation (4). Client proteins and a class of cochaperones, termed Hsp40 or J-domain proteins (JDPs), stimulate ATP hydrolysis on Hsp70, converting Hsp70 to the ADP state that binds client proteins with higher affinity and kinetic stability (1, 2, 3, 4). Studies with peptide substrates suggested that this client trapping is due in part to the closing of the α-helical lid over the peptide binding site in the SBD, although closing appears less pronounced with protein substrates (5, 6). Another class of cochaperones, the nucleotide exchange factors, facilitate client dissociation from Hsp70 by accelerating ADP release and, in some cases, directly contacting the Hsp70-SBD to drive client displacement (7, 8, 9).

JDPs have been reported to assist in Hsp70 function *via* two mechanisms that are mediated by distinct structural and functional domains. All JDPs contain a ∼70 residue J-domain that binds ATP-bound Hsp70 at the NBD–SBD interface and stimulates their ATPase activity. A conserved His-Pro-Asp (HPD) motif in the J-domain docks at the interdomain linker of Hsp70 to optimize two networks of hydrogen bonding and hydrophobic interactions at the ATPase active site (10) and is essential for stimulating ATP hydrolysis on Hsp70 (11, 12). In addition, two substrate-binding C-terminal domains (CTDI/CTDII) provide additional client interaction sites (13, 14). Although the role of the JDP/Hsp70 chaperone cycle in protecting and capturing client proteins is well established, whether their coupled chaperone cycle further modulates client conformation to promote folding has been a long-standing question. Recent works begin to address this question, showing that the bacterial JDP/Hsp70 homolog, DnaJ/K, can release luciferase in an altered conformation that folds more rapidly compared with that during spontaneous folding in dilute solution (15). Nevertheless, the generality of this phenomenon and whether JDPs participate in this client conformational modulation remain to be determined.

JDPs are divided into three classes (A, B, and C) based on the location of the J-domain and the presence of a zinc-finger-like region (16, 17, 18). Class A JDPs, such as *Escherichia coli* DnaJ and yeast Ydj1, contain an N-terminal J-domain, a glycine/phenylalanine-rich linker, CTDI/CTDII that provide client binding sites, and a C-terminal dimerization domain. Class B JDPs, represented by yeast Sis1, share the same domain organization as Class A JDPs, except that they lack the zinc-finger-like region insertion in the CTD-I of Class A JDPs. Class C JDPs contain additional specialized domains involved in diverse functions such as protein translation, pre-mRNA splicing, and clathrin-binding (19, 20, 21). Emerging evidence implicated all three classes of JDPs in an essential cellular process, the targeted delivery of nascent proteins to biological membranes (22, 23). The most abundant cytosolic JDPs, Ydj1 and Sis1, are required for the targeting of secretory proteins to the ER and the import of mitochondrial β-barrel and matrix proteins (24, 25). Ydj1 can bind a short cyclic β-hairpin on newly synthesized Tom40, suggesting that it plays a direct role in substrate recognition during targeting (25). Two additional cytosolic JDPs, Apj1 (Class A) and Jjj3 (Class C), were implicated in the targeting of glycosylphosphatidylinositol-anchored proteins to the ER (26). Finally, Xdj1 (Class A) and Djp1 (Class B) are required for the efficient assembly of the translocase of the outer mitochondrial membrane (27) and the biogenesis of the mitochondrial outer-membrane protein Mim1 (28), respectively. Despite the abundance of *in vivo* data, the mechanism by which cytosolic JDPs participate in the biogenesis of membrane and organellar proteins remains unclear.

We address this question in the guided entry of tail-anchored protein (GET) pathway, which delivers an essential class of tail-anchored membrane proteins (TAs) harboring hydrophobic transmembrane domains (TMDs) to the ER and is highly conserved across eukaryotic organisms. TAs contain a single TMD near the C terminus and mediate diverse cellular processes such as protein translocation across organelle membranes, vesicular trafficking, protein quality control, and apoptosis (29, 30). Recently, we demonstrated that newly synthesized TAs released from the ribosome are captured by the yeast cytosolic Hsp70, Ssa1, which protects hydrophobic TMDs from aggregation in the cytosol and is crucial for maintaining TAs in a translocation-competent state (31). Ssa1 further initiates a cascade of energetically downhill TA transfer events, first to the downstream cochaperone Sgt2 and then the targeting factor Get3 (31), which delivers TAs to the Get1/2 receptor complex at the ER for insertion into the membrane. Although the role of Ssa1 in the GET pathway has been established (31), whether and how additional Ssa1 cochaperones participate in TA targeting is unknown. In this work, we show that Ydj1 and Sis1, representing two different classes of JDPs, function redundantly to support the targeting of GET-dependent TAs *in vivo*. Unexpectedly, biochemical analyses showed that while Ydj1 assists Ssa1 in capturing TAs and preventing TA aggregation, Sis1 does not. On the other hand, both JDPs enhance the subsequent transfer of TA from Ssa1 to Sgt2. A functional J-domain in both JDPs was required to enhance the transfer competence of Ssa1-bound TA and to support efficient TA insertion *in vivo*. Our results uncover a new role of JDPs in regulating the conformation of Hsp70-bound client proteins to enhance their targeting competence.

## Results

### Ydj1 and Sis1 are essential for TA insertion into the ER *in vivo*

**Figure 1:**
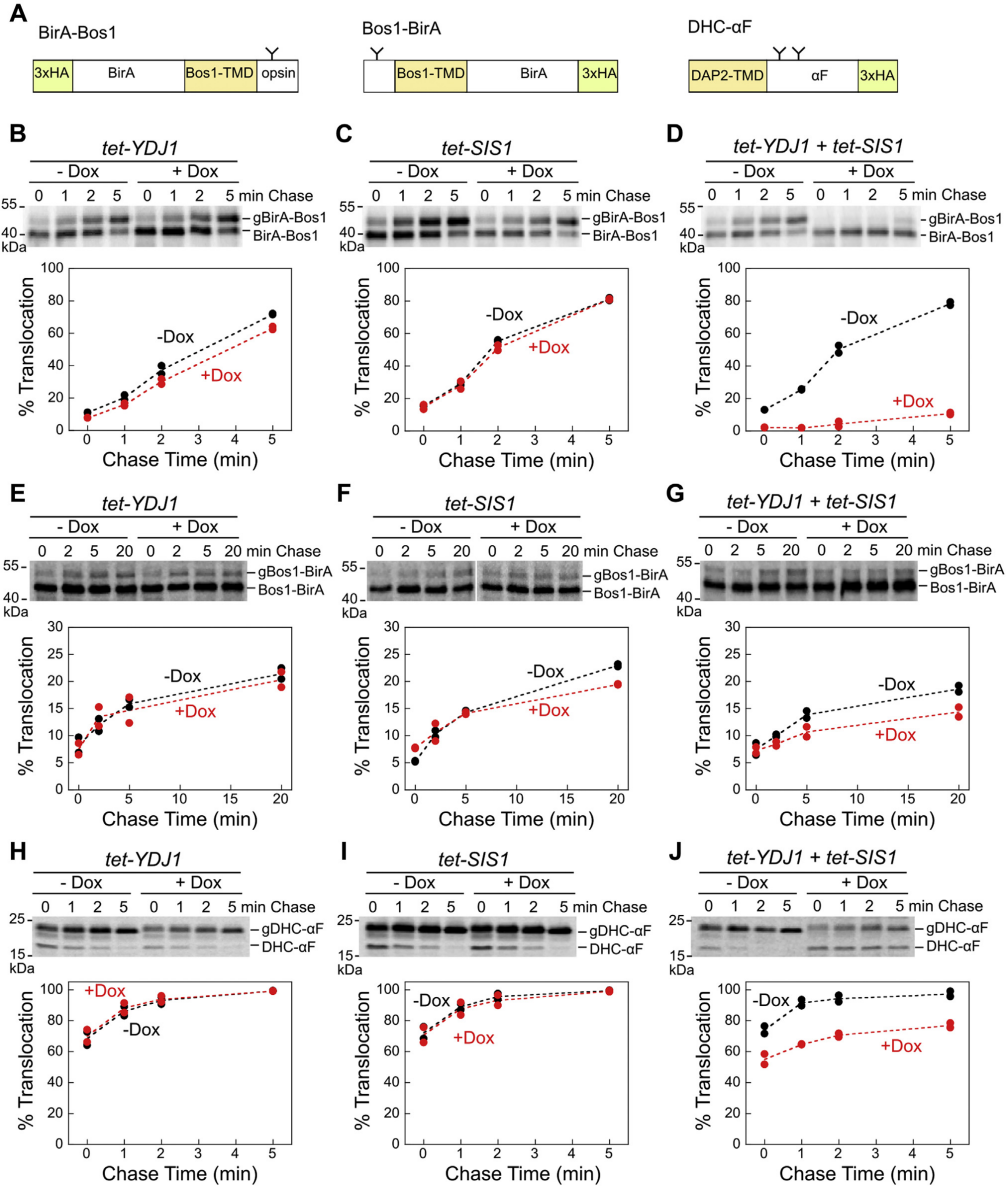
**Depletion of both Ydj1 and Sis1 abolish the ER targeting of TAs *in vivo*.***A*, scheme of model substrates used in the *in vivo* targeting assay (31). “Y” denotes the glycosylation sites that allow detection of substrate insertion into the ER in the correct topology. N- or C-terminal 3xHA tags were used for immunoprecipitation of the substrates. *B–J*, *top*, representative autoradiograms for pulse-chase analysis of the translocation of metabolically labeled BirA-Bos1 (*B–D*), Bos1-BirA (*E–G*), and DHC-αF (*H–J*) in *tet-YDJ1*, *tet-SIS1*, and *tet-YDJ1/tet-SIS1* cells in the absence (−) and presence (+) of Doxycycline (Dox). “g” denotes glycosylated substrates, which migrate more slowly than nonglycosylated substrates. *Bottom*, quantification of the data in (*B–J*) and their replicates. Values from two biological replicates are shown as *black circles* (−Dox) and *red circles* (+Dox). TA, tail-anchored protein.

We first asked whether cytosolic JDPs are involved in the ER targeting of TAs. Because two JDPs, Ydj1 and Sis1, were implicated in the translocation of α factor into the ER and the import of mitochondrial precursor proteins (24, 25), we hypothesized that they also assist Ssa1 in TA targeting to the ER membrane *in vivo*. To test this hypothesis, we used an established set of isogenic yeast strains in which the expression of YDJ1, SIS1, or both genes are under the control of a tetracycline-repressible promoter (*tet-YDJ1*, *tet-SIS1*, and *tet-YDJ1/tet-SIS1*; (25)). We depleted Ydj1 and/or Sis1 in these strains using 4 h of doxycycline treatment (Fig. S1), as described (25), and carried out pulse-chase assays to measure the insertion kinetics of newly synthesized model substrates into the ER (Fig. 1*A*). We used BirA fused to the C-terminal TMD of the SNARE protein Bos1 (BirA-Bos1) as the model TA for the GET pathway, because it is strongly dependent on Ssa1 and Get3 for insertion into the ER (31). Successful insertion leads to efficient glycosylation of an opsin tag at the C terminus of BirA-Bos1 (Cho and Shan, 2018), providing a quantitative readout for targeting and translocation efficiency. We found that depletion of either Ydj1 or Sis1 did not significantly affect the insertion efficiency of BirA-Bos1 (Fig. 1, *B* and *C*), whereas the depletion of both nearly abolished BirA-Bos1 insertion (Fig. 1*D*).

To exclude the possibility that the observed translocation defect of BirA-Bos1 arose from pleiotropic effects in *tet-YDJ1/tet-SIS1* cells, such as a reduced cytosolic folding capacity or compromised ER membrane, we tested two control substrates, Bos1-BirA and DHC-αF, whose targeting and translocation are independent of Hsp70 and Get3 (Fig. 1*A*) (31). In Bos1-BirA, the Bos1-TMD is upstream of the BirA moiety and near the N terminus of the protein, making it a substrate for the co-translational signal recognition particle (SRP) pathway (31). In DHC-αF, the N-terminal signal sequence of prepro-α factor is replaced by the TMD of aminopeptidase B (DAP2) to convert it into an SRP-dependent substrate that uses the co-translational targeting pathway (32). Pulse-chase experiments showed that depletion of both Ydj1 and Sis1 had only modest effects on the translocation of Bos1-BirA (Fig. 1, *E*–*G*) and DHC-αF (Fig. 1, *H–J*), suggesting that nonspecific folding defects are insufficient to account for the large deleterious effects of the Ydj1/Sis1 depletion on the ER insertion of BirA-Bos1.

It has been reported that yeast harbors multiple alternative targeting pathways to mediate post-translational membrane protein targeting to the ER (33). For example, the SRP-independent (SND) pathway mediates the targeting of proteins with an internal TMD (34), and TAs with low hydrophobicity TMDs are targeted by GET-independent pathways (35, 36). To test whether the Hsp70/JDP system functions in these alternative pathways, we used two established substrates, BirA-6AG (35) and Scs2-GFP (34) (Fig. S2*A*). BirA-6AG is a GET-independent TA in which multiple hydrophobic residues in the Bos1 TMD are replaced by Ala/Gly to reduce hydrophobicity (Fig. S2*B*) (35). Scs2-GFP is an established SND substrate containing an internal TMD (34). We tested the Ydj1/Sis1-dependence for the translocation of both substrates using the *tet-YDJ1/tet-SIS1* cells as above. We also tested the Hsp70-dependence using the established SSA1 (*SSA1ssa2Δssa3Δssa4Δ*) and *ssa1*^*ts*^ (*ssa1*^*ts*^*ssa2Δssa3Δssa4Δ*) strains, in which a temperature-sensitive mutant Ssa1 in *ssa1*^*ts*^ is rapidly inactivated within 5 min upon shift to nonpermissive temperature (37). While the ER targeting of both BirA-6AG and Scs2-GFP proteins are independent of Get3 (Fig. S2*C*), the translocation efficiencies of both substrates were reduced substantially upon transient inactivation of Ssa1 or depletion of Ydj1/Sis1 (Fig. S2, *D–G*). These results strongly suggest that the cytosolic Hsp70/JDP system, comprised of Ydj1/Sis1 and Ssa1, is involved in the post-translational ER targeting of multiple classes of membrane proteins in yeast.

Together, these results show that two cytosolic JDPs, Ydj1 and Sis1, function redundantly to facilitate the post-translational targeting of TAs to the ER *in vivo*. The strong TA insertion defect upon depletion of both JDPs further indicate that at least one of them is required for the efficient targeting of GET-dependent substrates and that this role cannot be filled by other JDPs. Finally, this JDP/Hsp70 system is also required for the efficient targeting of GET-independent TAs and SND substrates, suggesting that they form a chaperone hub upstream of diverse post-translational membrane protein targeting pathways in yeast.

### Ydj1 and Sis1 differ in their ability to assist Hsp70 in substrate trapping

**Figure 2:**
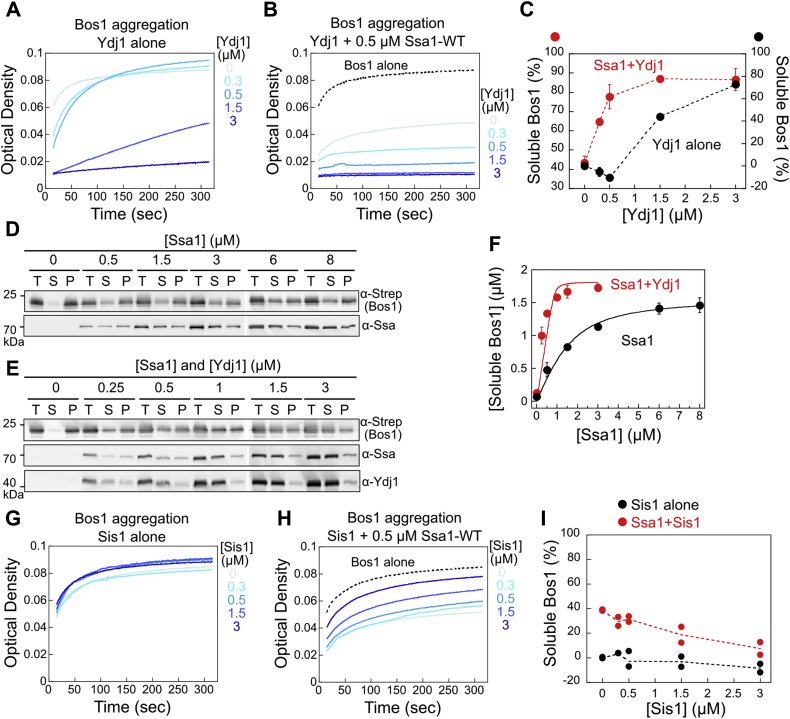
**Ydj1 cooperates with Ssa1 to suppress Bos1 aggregation *in vitro*, but Sis1 does not.***A* and *B*, time courses of Bos1 aggregation in the presence of indicated concentrations of Ydj1 without (*A*) and with (*B*) 0.5 μM Ssa1 present. *C*, quantification of the data in (*A*), (*B*), and their replicates. Note the different *y*-axis scales for the data with and without Ssa1. *D* and *E*, representative Western blot analyses of sedimentation experiments to measure the solubility of 3 μM Bos1 in the presence of Ssa1 (*D*) or equimolar mixtures of Ssa1 and Ydj1 (*E*) at the indicated concentrations. “T”, “S”, and “P” represent total input, soluble, and pellet, respectively. *F*, quantification of the concentration of soluble Bos1 from the data in (*D*), (*E*), and their replicates. The data were fit to Equation 2, which gave *K*_Soluble_ values of 0.73 ± 0.012 μM with Ssa1 and 0.014 ± 0.003 μM with both Ssa1 and Ydj1 present. *G* and *H*, time courses of Bos1 aggregation in the presence of indicated concentrations of Sis1 without (*G*) and with (*H*) 0.5 μM Ssa1 present. *I*, quantification of the data in (*G*), (*H*), and their replicates. All values in (*C*) and (*F*) are reported as mean ± SD, with n = 3. Error bars are shown but may not be visible in some cases. The values from two independent experiments are shown in (*I*) as *black* (Ssa1 alone) and *red* (Ssa1+Sis1) *circles*.

We investigated the molecular mechanisms by which Ydj1 and Sis1 facilitate the ER targeting of TAs in biochemical analyses. As Ssa1 is required to capture nascent TAs in the soluble form (31), and as JDPs have been suggested to autonomously bind substrates and to assist Hsp70s in substrate trapping (10, 14, 38, 39), we first asked whether Ydj1 and Sis1 enhance the ability of Ssa1 to capture TAs and prevent them from aggregation. As the model TA substrate *in vitro*, we used a noncleavable, soluble SUMO domain fused to the Bos1 TMD (termed Bos1) (31, 35, 40). Dilution of purified, detergent-solubilized Bos1 into aqueous buffer, which removes detergent micelles, led to rapid aggregation of Bos1 as monitored by the turbidity assay (Fig. 2*A*, *light blue line*). Using this assay, we confirmed that ATP-bound Ssa1 effectively prevented Bos1 aggregation (Figs. S3*A* and S1*B*, *red*), as reported (31). In comparison, ADP-bound Ssa1 prevented TA aggregation less efficiently (Fig. S3, *A* and *B*, *green* *versus* *red*), and apo-Ssa1 was unable to suppress TA aggregation (Fig. S3, *A* and *B*, *blue*). These results show that nucleotide binding is required for Ssa1 to capture TAs in the soluble form, and that ATP-bound Ssa1 is most efficient in TA capture.

Ydj1 by itself only prevented Bos1 aggregation at super-stoichiometric concentrations (≥3 μM Ydj1 *versus* 1.5 μM Bos1; Fig. 2*A*). In comparison, 0.5 μM Ssa1 prevented the aggregation of ∼50% Bos1 (Fig. 2*B*, *dotted black* *versus* *light blue lines*), as reported (31), indicating that Ssa1 is a more effective chaperone than Ydj1 for Bos1. In the presence of 0.5 μM Ssa1, addition of substoichiometric amounts of Ydj1 significantly enhanced the solubility of Bos1 (Fig. 2, *B* and *C*, *red*), suggesting that Ydj1 cooperates with Ssa1 to prevent Bos1 aggregation. To better estimate the efficacy of the Ydj1·Ssa1 complex in TA capture, we used a sedimentation-based assay that detects both the soluble (supernatant) and insoluble (pellet) fractions of Bos1 (Fig. 2, *D* and *E*). In the absence of Ydj1, Ssa1 generated a maximum of 1.5 μM soluble Bos1 at saturating concentrations, and >3 μM Ssa1 was required for saturation (Fig. 2*F*, *black circles*). With equimolar Ydj1 and Ssa1 present, the maximum amount of soluble TA substrate was similar (1.7 *versus* 1.5 μM), but saturation occurred at lower Ssa1 concentrations (Fig. 2*F*, *red circles*). Analysis of the chaperone concentration dependences of the sedimentation data yielded apparent constants (*K*_*soluble*_) of 0.014 ± 0.003 and 0.73 ± 0.012 μM, respectively, for the chaperoning of Bos1 by Ssa1 with and without Ydj1. These results provide independent evidence for the synergy between Ydj1 and Ssa1 in the capture and maintenance of soluble TA.

In contrast to Ydj1, Sis1 displayed no autonomous chaperone activity toward Bos1 (Fig. 2*G*). In the presence of 0.5 μM Ssa1, the addition of Sis1 reduced rather than further improved the solubility of Bos1 (Fig. 2, *H* and *I*). These observations are consistent with the previously reported difference in the client binding domains and properties of Ydj1 and Sis1 (41, 42). Importantly, the inability of Sis1 to chaperone TAs either by itself or in concert with Ssa1 strongly suggests that these activities are not required for its role in facilitating TA insertion into the ER *in vivo* (Fig. 1). Thus, the roles of JDPs in supporting TA insertion are unlikely to be solely attributed to enhanced TA capture by the Ssa1/JDP pair.

### Ydj1 and Sis1 enhance TA transfer from Ssa1 to Sgt2

**Figure 3:**
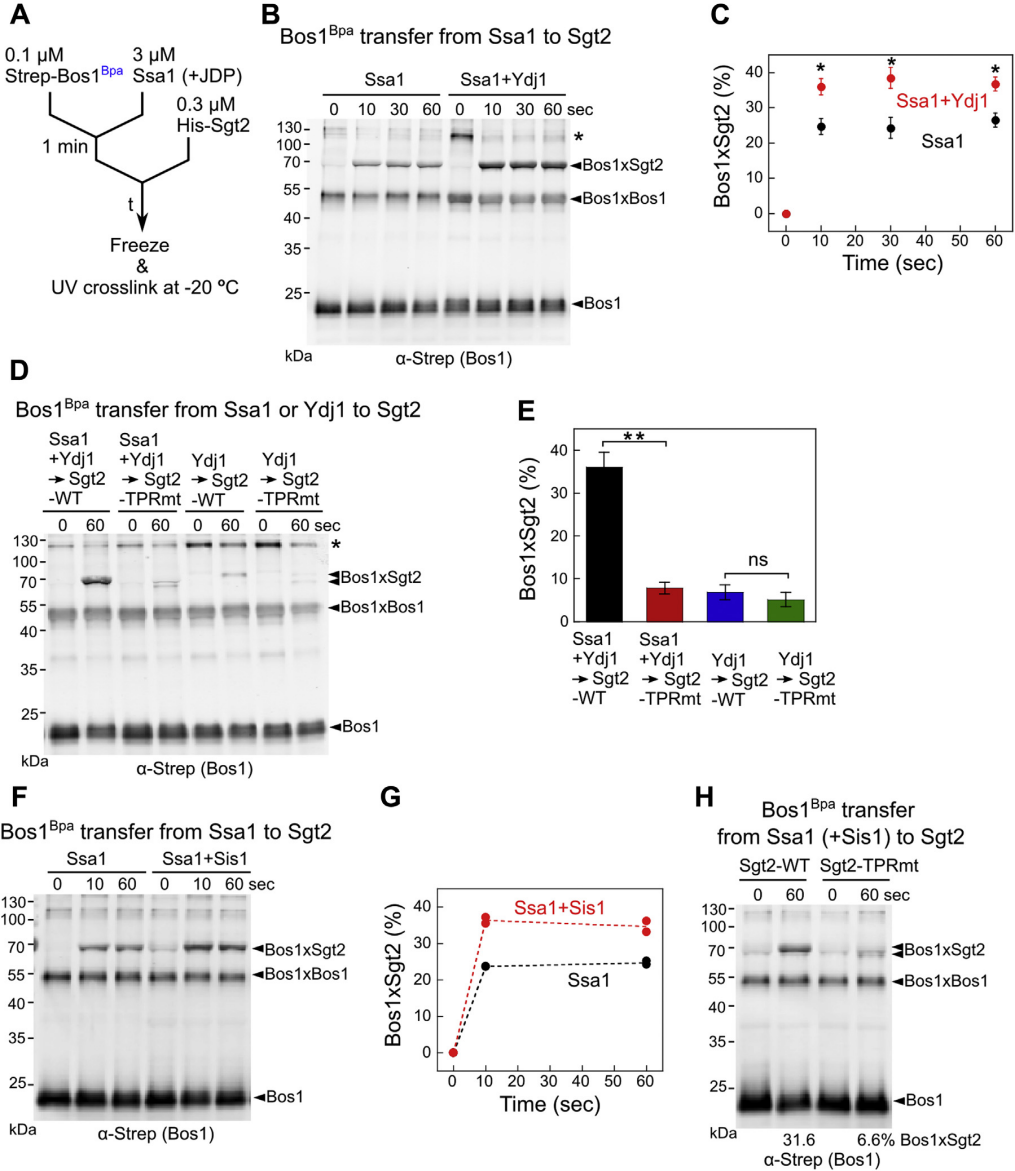
**Ydj1 and Sis1 enhance TA transfer from Ssa1 to Sgt2.***A*, scheme of the assay to measure Bos1^Bpa^ transfer from Ssa1 to Sgt2. 0.1 μM Bos1^Bpa^ was preincubated with 3 μM Ssa1 and/or JDP, followed by addition of 0.3 μM Sgt2 to initiate the transfer reaction. At specified times after Sgt2 addition (t), aliquots of the reaction were frozen and analyzed by UV crosslinking at –20 °C. *B*, representative Western blot analysis of the time courses of Bos1^Bpa^ transfer from Ssa1 to Sgt2 in the absence and presence of Ydj1. *C*, quantification of the Bos1-Sgt2 crosslink from the data in (*B*) and their replicates. *D*, representative Western blot image of Bos1^Bpa^ transfer from Ydj1 alone or Ssa1·Ydj1 to Sgt2 and Sgt2-TPRmt. Two crosslinked bands were observed between Bos1 and Sgt2-TPRmt, possibly due to different conformations of the Bos1·Sgt2-TPRmt complex. *E*, quantification of the efficiency of Bos1-Sgt2 crosslinking from the data in (*D*) and their replicates. *F*, representative Western blot analysis of the time courses of Bos1^Bpa^ transfer from Ssa1 to Sgt2 in the absence and presence of Sis1. *G*, quantification of the Bos1-Sgt2 crosslink from the data in (*F*) and their replicates. *H*, representative Western blot image of Bos1^Bpa^ transfer from Ssa1 and Sis1 to Sgt2 and Sgt2-TPRmt. All values in (*C*) and (*E*) represent mean ± SD, with n ≥ 3; “∗” and “∗∗” denote *p* < 0.005 and *p* < 0.001, respectively, from Student's *t* test. Error bars are shown but may not be visible in some cases. Values from two independent experiments are shown in (*G*) as *black* (Ssa1 alone) and *red* (Ssa1+Sis1) *circles*. TA, tail-anchored protein.

The only other major step involving Ssa1 in the GET pathway is the transfer of its bound TA to the cochaperone Sgt2 (31). To test if the JDPs regulate this step, we monitored TA transfer using a photocrosslinker, p-benzoyl-l-phenylalanine (Bpa), site-specifically incorporated into the eighth residue (Ile) in the Bos1-TMD using amber suppression (43). Purified, Bpa-incorporated Bos1 (Bos1^Bpa^) was preincubated with Ssa1 for 1 min to form the soluble Ssa1·Bos1^Bpa^ complex, followed by the addition of His_6_-tagged Sgt2 to initiate TA transfer (Fig. 3*A*). Before and at different times during the transfer, aliquots of the reaction were flash frozen, and the chaperone association of Bos1^Bpa^ was monitored by UV-induced photocrosslinking (Figs. 3*A* and S4). Both Ssa1 and Ydj1 form ∼125 kDa crosslinks to Bos1^Bpa^ (Fig. S4, *A–E*). Compared with the Bos1-Ydj1 crosslink, the Bos1-Ssa1 crosslink was weak and diffuse (Fig. S4, *A–E*) despite the more effective TA capture by Ssa1 than Ydj1 (Fig. 2 and (31)), likely reflecting the more dynamic nature of the TA interaction with Ssa1. In contrast, the Bos1-Sgt2 crosslink at ∼70 kDa was distinct and readily detectable (Figs. 3*B* and S4). We therefore monitored the efficiency of the Ssa1-to-Sgt2 TA transfer based on the Bos1-Sgt2 crosslink.

Upon the addition of Sgt2, the Bos1-Ssa1 crosslink at ∼125 kDa disappeared and was replaced by a crosslinked band to Sgt2 (Fig. S4, *B* and *C*), indicating the transfer of TA from Ssa1 to Sgt2. Consistent with the results of fluorescence-based TA transfer assays (31), the Bos1-Sgt2 crosslink was substantially reduced by the R171A, R175A mutations on the conserved tetratricopeptide repeat (TPR) domain of Sgt2 (TPRmt), which disrupts Sgt2 binding with Ssa1 (40), corroborating that the transfer was strongly dependent on the Ssa1-Sgt2 interaction (Figs. 3*C* and S5, *A–C*; (31)).

We asked if Ydj1 participates in the TA transfer event. Time course measurements showed that TA transfer from Ssa1 to Sgt2 was rapid and complete within 10 s both with and without Ydj1 present (Figs. 3*B* and S5). Compared to the Ssa1·Bos1 complex formed without Ydj1, the presence of Ydj1 during the preincubation of Ssa1 with TA significantly increased the fraction of TA substrate crosslinked to Sgt2 (36.8 ± 1.1% *versus* 26.5 ± 2.2%) (Fig. 3, *B* and *C*). We excluded the possibility that this enhancement was due to the presence of an additional pathway in which Ydj1 directly transfers its bound TA to Sgt2. First, the transfer reaction with both Ssa1 and Ydj1 present is abolished by the R171A, R175A mutations on the conserved TPR domain of Sgt2 (TPRmt) that disrupt its binding with Ssa1 (40) (Fig. 3, *D* and *E*). Thus, the transfer reaction in the presence of Ydj1 is still dependent on the interaction of Ssa1 with the Sgt2 TPR domain, as was expected for a direct Ssa1-to-Sgt2 TA transfer (Fig. S5, *A–C*; (31)). In addition, only low levels of Bos1-Sgt2 crosslink were detected when Bos1 premixed with Ydj1 was incubated with Sgt2, and the Sgt2(TPRmt) did not affect direct TA transfer from Ydj1 to Sgt2 (Fig. 3, *D* and *E*, *blue* and *green*). Thus, even in the presence of Ydj1, the Ssa1-to-Sgt2 transfer is the dominant pathway for TA loading on Sgt2, and this transfer was enhanced by Ydj1.

The following observations indicate that this enhancement is distinct from the roles of Ydj1 in assisting the capture of soluble TA. First, a saturating Ssa1 concentration (3 μM) was used during the preincubation with TA before initiation of transfer, bypassing the Ydj1-mediated enhancement of TA binding by Ssa1. Second, when we changed the order of addition and supplemented Ydj1 to Sgt2 during the transfer phase after Bos1 was preloaded on Ssa1, the enhancement in the Bos1–Sgt2 crosslink persisted (Fig. S6). This ruled out models in which a pool of TA substrates was irreversibly trapped in transfer-incompetent conformations if the initial TA capture occurred without Ydj1. Third, as shown in the next section, the effects of Ydj1 on the TA capture and transfer steps have distinct molecular requirements and can be experimentally uncoupled. Finally, the same enhancement in Ssa1-to-Sgt2 TA transfer was observed with Sis1. Despite the inability of Sis1 to chaperone the TA substrate or to assist Ssa1 in TA trapping (Fig. 2, *H* and *I*), the presence of Sis1 significantly increased the fraction of Bos1^Bpa^ crosslinked to Sgt2 after the transfer (Fig. 3, *F* and *G*), with efficiency comparable to that in the presence of Ydj1. The enhanced TA transfer from Ssa1·Sis1 to Sgt2 is also dependent on the interaction of Ssa1 with the Sgt2 TPR domain (Fig. 3*H*), supporting a direct TA transfer from Ssa1 to Sgt2 in the presence of Sis1. Together, these data strongly suggest an additional regulatory role of JDPs in enhancing the transfer competence of Ssa1-bound TA to Sgt2, thus committing the TA to localization at the ER membrane.

### A functional J-domain is required to enhance the transfer competence of Ssa1-bound TA

**Figure 4:**
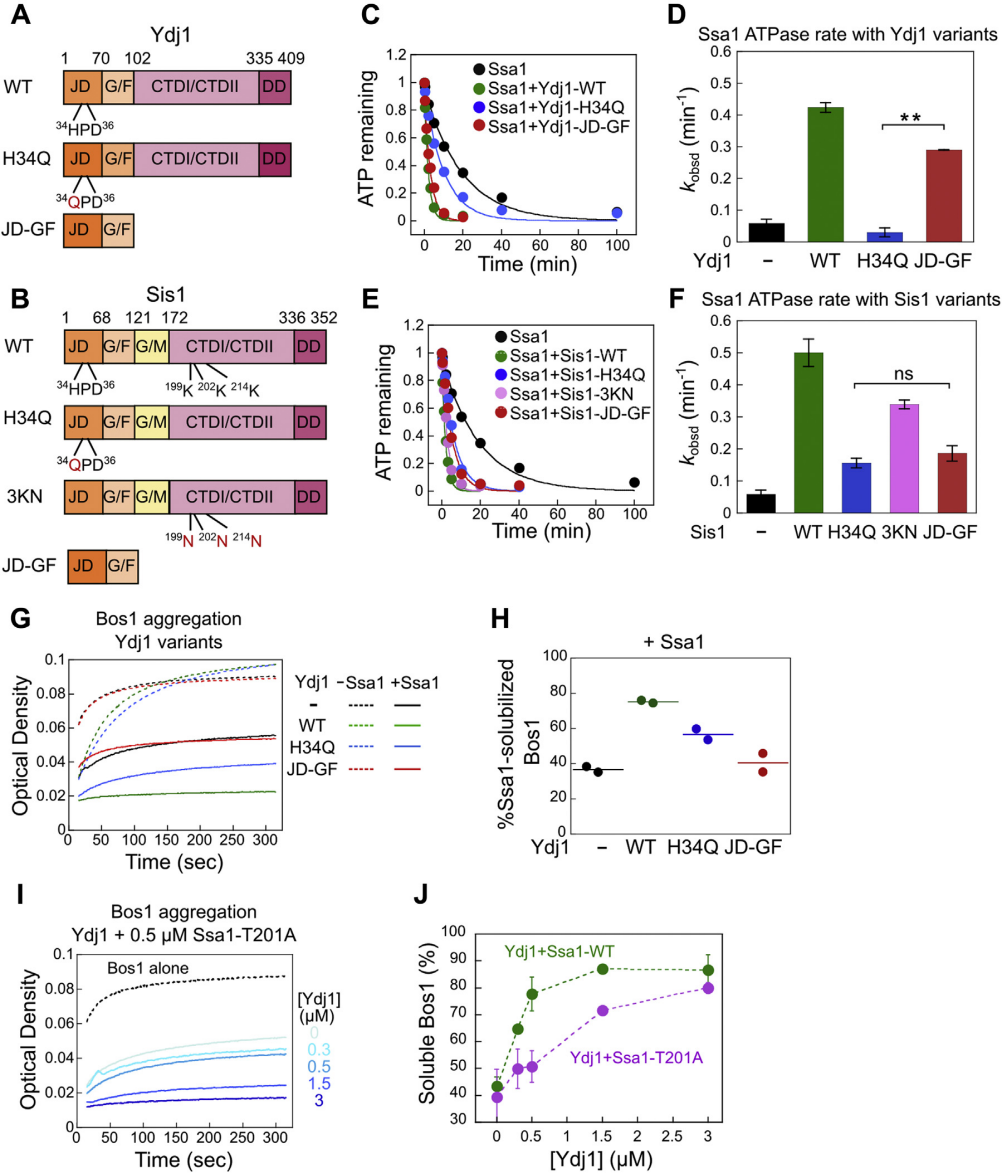
**Ydj1 assists Ssa1 in TA capture in a soluble form using both its CTDs and J-domain.***A* and *B*, schematic of the domain organization of wildtype and mutant Ydj1 (*A*) and Sis1 (*B*) tested in this work. *C–F*, representative time courses for single-turnover ATP hydrolysis reactions were shown for reactions with 3 μM Ssa1 in the absence and presence of 3 μM wildtype and mutant Ydj1 (*C*) or Sis1 (*E*). ATPase reactions were measured and analyzed as described under Experimental procedures. Lines are fits of the data to Equation 3, and the obtained ATPase rate constants are summarized in parts (*D*) and (*F*). *G*, time courses of Bos1 aggregation in the presence of 0.5 μM of the indicated Ydj1 variants with (*solid lines*) and without (*dotted lines*) 0.5 μM Ssa1. H, the difference in optical density between the (−Ssa1) and (+Ssa1) reactions in (*G*) and their replicates are quantified. The *lines* represent the mean from two independent experiments. *I*, time courses of Bos1 aggregation in the presence of 0.5 μM Ssa1(T201A) and indicated concentrations of Ydj1. *J*, quantification of the data in (*I*) and their replicates (*purple*). The data for 0.5 μM Ssa1-WT and Ydj1 (*green*) were from Figure 2*C* and shown for comparison. All values in (*D*), (*F*), and (*J*) are reported as mean ± SD, with n ≥ 3. “∗∗” denotes *p* < 0.001 from Student's *t* test. Error bars are shown but may not be visible in some cases. CTDI/CTDII, C-terminal domains I and II; DD, dimerization domain; G/F, Glycine and Phenylalanine-rich linker; G/M, Glycine and Methionine-rich region; JD, J-domain; TA, tail-anchored protein.

To decipher how JDPs cooperate with Ssa1 in the GET pathway, we tested mutations that block their distinct activities (Fig. 4, *A* and *B*). The H34Q mutation in the conserved HPD motif of Ydj1 and Sis1 abolishes the ability of the J-domain to activate ATP hydrolysis in Hsp70 (Fig. 4, *C*–*F*) but does not affect client-binding in CTDI/CTDII (11, 13, 39). JD-GF contains the JD and glycine/phenylalanine linker of Ydj1 or Sis1, which binds and regulates Hsp70, but lacks the CTDI/CTDII that bind client proteins. Ydj1(JD-GF) activated the ATPase reaction of Ssa1 ∼5-fold, only 30% lower than wildtype Ydj1, whereas ATPase activation by Sis1(JD-GF) was comparable to Sis1(H34Q) (Fig. 4, *C*–*F*). These results suggest that Ydj1 JD-GF is necessary and sufficient for ATPase activation of Ssa1, whereas this activation requires participation by additional domains of Sis1. Mutation of three conserved lysines in Sis1-CTDI (K199/201/214N, or 3KN) disrupts the binding of CTDI with the EEVD motif of Ssa1 (44), but largely preserved the ATPase activation of Ssa1 (Fig. 4, *C*–*F*). These mutants allow us to uncouple the functions of JDPs in client-binding and Hsp70 regulation. Finally, we introduced the T201A mutation in the Ssa1 ATPase site. Mutation of the homologous residue (T199A) in *E. coli* DnaK reduced the ATP hydrolysis rate without affecting ATP binding (45). We further confirmed that Ssa1(T201A) nearly abolished the Ydj1 and Sis1-mediated ATPase activation of Ssa1 (Fig. S7, *A* and *B*). This mutant allowed us to assess whether ATPase activation on Ssa1 is required for the JDP-mediated regulations.

Using these mutants, we asked which domain(s) was used by Ydj1 to cooperate with Ssa1 during TA capture (Fig. 4, *G* and *H*). In the absence of Ssa1, Ydj1(H34Q) modestly delayed the aggregation of Bos1, similar to observations with wildtype Ydj1, whereas Ydj1(JD-GF) had no effect on Bos1 aggregation, as expected from the lack of client binding domains (Fig. 4*G*, dotted lines). In the presence of equimolar Ssa1, Ydj1(H34Q) was less efficient than wildtype Ydj1 in helping to suppress the aggregation of Bos1 (Fig. 4*G*, solid lines and Fig. 4*H*). Ydj1 was also less efficient in assisting TA capture by mutant Ssa1(T201A) (Fig. 4, I and *J*, also see Fig. 2, *A*–*C*), which abolishes the JDP-induced ATPase activation of Ssa1 (Fig. S7*B*), as does Ydj1(H34Q). In contrast, the T201A mutation did not affect the chaperone activity of Ssa1 toward the TA in the absence of Ydj1 (Fig. S8, *A* and *B*). These observations indicate that the J-domain–mediated ATPase activation of Ssa1 plays an important role in the cooperation of Ydj1 with Ssa1 during TA capture. Ydj1(JD-GF) abolished the synergy with Ssa1 in preventing Bos1 aggregation (Fig. 4, *G* and *H*, red), indicating that client binding by the Ydj1 CTDI/CTDII is required for Ydj1 to assist Ssa1 during TA trapping. Finally, none of the Sis1 variants assisted Ssa1 in TA trapping, as expected from the lack of this activity with wildtype Sis1 (Fig. S8, *C* and *D*).

**Figure 5:**
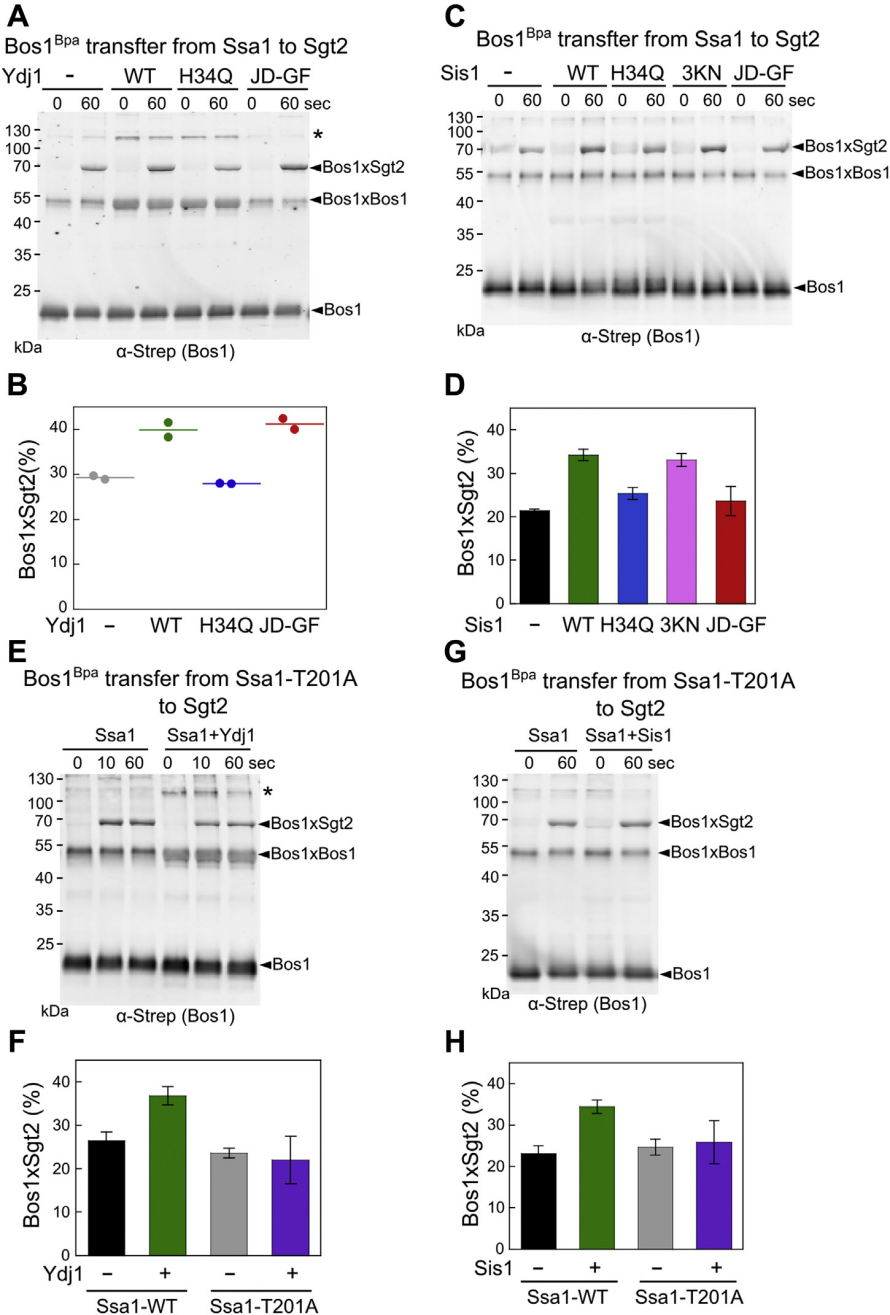
**A functional J-domain in Ydj1 and Sis1 is required for enhancing the Ssa1-to-Sgt2 TA transfer.***A* and *C*, representative Western blot images of Bos1^Bpa^ transfer from Ssa1 to Sgt2 in the presence of the indicated Ydj1 (*A*) or Sis1 (*C*) variants. Reactions were carried out as described in Figure 3*A*. *B* and *D*, quantification of the efficiency of the Bos1-Sgt2 crosslink from replicates of the data in (*A*) and (*C*), respectively-. *E* and *G*, representative Western blot image of the time courses of Bos1^Bpa^ transfer from Ssa1(T201A) to Sgt2 in the absence and presence of Ydj1 (*E*) or Sis1 (*G*). *F* and *H*, quantification of the efficiency of the Bos1-Sgt2 crosslink from replicates of the data in (*E*) and (*G*), respectively. All values in (*D*), (*F*), and (*H*) are reported as mean ± SD, with n ≥ 3. Error bars are shown but may not be visible in some cases. The *lines* in (*B*) represent the mean from two independent measurements. TA, tail-anchored protein.

We further dissected the functional requirement of JDPs during the Ssa1-to-Sgt2 TA transfer. Despite its inability to bind Bos1 or help Ssa1 in TA capture (Fig. 4, *G* and *H*), Ydj1(JD-GF) was sufficient to enhance the fraction of Bos1 crosslinked to Sgt2, with efficiencies comparable to that of Ydj1-WT (41.2 ± 1.7% and 39.9 ± 2.3%, respectively; Fig. 5, *A* and *B*). In contrast, Ydj1(H34Q) did not alter the transfer efficiency of Bos1 (Fig. 5, *A* and *B*). These results indicate that the interaction and regulation of Ssa1 by the Ydj1 J-domain is necessary and sufficient for enhancing the Ssa1-to-Sgt2 TA transfer. Both Sis1 mutants H34Q and JD-GF significantly reduced the Sis1-induced enhancement of TA transfer from Ssa1 to Sgt2, whereas mutant Sis1(3KN) stimulated the transfer almost as well as WT Sis1 (Fig. 5, *C* and *D*). Importantly, the enhancement of TA transfer efficiencies observed with the Ydj1 and Sis1 variants strongly correlated with their ability to activate ATP hydrolysis of Ssa1 (Fig. 4, *B*–*F*). Finally, Ssa1(T201A) abolished the ability of both JDPs to enhance the Bos1-Sgt2 crosslink after TA transfer (Fig. 5, *E*–*H*), although this mutation did not affect TA transfer in the absence of the JDPs (Fig. S5, *A*–*C*). This provides additional evidence that the JDP-stimulated ATP hydrolysis in Ssa1 is required for the enhancement of TA transfer. Together, these data show that both Ydj1 and Sis1 use their J-domains to regulate the nucleotide state and conformation of the Ssa1·Bos1 complex, thus enabling more efficient TA transfer to Sgt2. In contrast, the client binding domains of Ydj1 are dispensable for this process, providing further evidence that the enhancement of the Ssa1-to-Sgt2 TA transfer by Ydj1 can be uncoupled from its role in assisting Ssa1 in TA capture and preventing TA aggregation.

### A functional J-domain is required for JDPs to support efficient TA insertion *in vivo*

**Figure 6:**
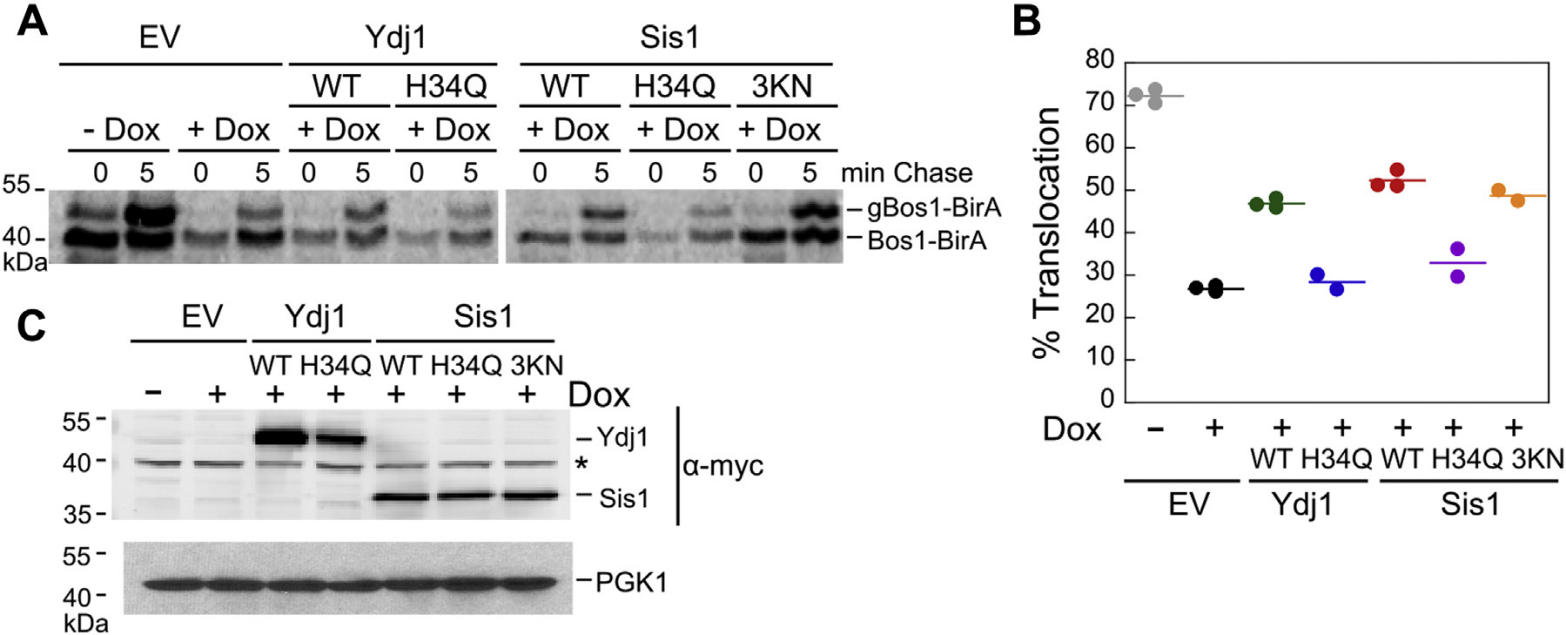
**A functional J-domain in Ydj1 and Sis1 is required for the ability of these JDPs to support efficient TA insertion *in vivo*.***A*, a representative autoradiogram for pulse-chase analysis of the translocation of metabolically labeled BirA-Bos1 upon expressing empty vector (EV) or the indicated Ydj1 and Sis1 variants. *B*, quantification of the data in (*A*) and their replicates. The *lines* represent the mean from two or three biological replicates, as indicated. *C*, anti-myc Western blot to detect the expression levels of Ydj1 and Sis1 variants in the indicated cell lysate. PGK1 serves as a loading control. JDP, J-domain protein; TA, tail-anchored protein.

To test which domain(s) and activities of JDP are important for assisting TA insertion *in vivo*, we carried out complementation analyses. After depletion of both Sis1 and Ydj1 in the *tetYDJ1/tet-SIS1* strain by doxycycline treatment as described earlier (25), we induced the expression of myc-tagged Ydj1, Sis1, or their variants and measured the ER insertion of BirA-Bos1 using pulse-chase assays. At 5 min after the chase, depletion of Ydj1 and Sis1 reduced the insertion of BirA-Bos1 from ∼72% to 27% (Fig. 6, *A* and *B*). Overexpression of WT Ydj1 rescued TA insertion to 47%, whereas the Ydj1(H34Q) mutant provided no rescue compared with the empty vector (EV) control (Fig. 6, *A* and *B*). Despite its inability to assist Ssa1 in TA capture (Fig. 2, *H* and *I*) and its lower expression levels compared with Ydj1 (Fig. 6*C*), expression of Sis1 rescued TA insertion slightly more effectively than Ydj1 (Fig. 6, *A* and *B*). Sis1(H34Q) reduced the rescue to levels comparable to the EV control, whereas Sis1(3KN) rescued the insertion of BirA-Bos1 as effectively as WT Sis1 (Fig. 6, *A* and *B*). Anti-myc Western blot showed comparable expression levels for Sis1, Sis1(H34Q), and Sis1(3KN) (Fig. 6*C*), indicating that the different rescues observed with WT and mutant Sis1 reflect differences in *in vivo* activity rather than their abundance. Although Ydj1(H34Q) was expressed at a lower level than WT Ydj1, the abundance of Ydj1(H34Q) was comparable to those of WT and mutant Sis1, suggesting that the failure of Ydj1(H34Q) to rescue the ER insertion of BirA-Bos1 was not because of insufficient protein expression levels. Together, these results show that a functional J-domain in Ydj1 and Sis1 to regulate Hsp70 activity is required for the ability of these JDPs to support efficient TA insertion.

## Discussion

The effectiveness and versatility of Hsp70 function rely on its cooperation with JDPs, which drive diverse and specific functions of Hsp70s (22, 23, 46). The results from this and a recent work (25) showed that among the 13 yeast cytosolic JDPs, two JDPs from distinct classes, Ydj1 and Sis1, serve redundant roles in promoting the targeted delivery of multiple classes of membrane proteins, including the targeting of GET- and GET-independent substrates to the ER (this work) and the import of β-barrel proteins to mitochondria (25). Further, biochemical analyses in the GET pathway uncovered a new activity of JDPs in enhancing the transfer competence of Hsp70-bound TAs to downstream chaperones. These findings provide evidence that in addition to their previously established roles in facilitating client capture on Hsp70s, JDPs enable Hsp70 to bind client proteins in a conformation more conducive to their successful biogenesis.

The ability of JDPs to assist in client trapping on Hsp70 is well studied (38, 39, 47, 48) and typically occurs *via* two mechanisms. First, the J-domain activates ATP hydrolysis on Hsp70, converting the latter to the ADP-bound state that binds substrates more tightly (4, 49, 50). Second, some JDPs contain client-binding motifs that can also bind aggregation-prone proteins (38, 39, 47, 48). Our results showed that Ydj1 uses both mechanisms to assist Ssa1 in capturing and maintaining the solubility of newly synthesized TAs. Nevertheless, this activity is not shared by Sis1, a class B JDP. These findings are consistent with the differences between class A and class B JDPs reported previously. Multiple peptide binding sites have been detected in Ydj1's CTDI, CTDII, and zinc-finger motif, which provide redundant interactions for client binding, whereas Sis1 and other type B JDPs contain only one peptide binding site in CTDI (13, 41, 51). Moreover, peptide binding in Sis1 is displaced by the EEVD motif of Hsp70, whereas peptide interaction with Ydj1 persists in the presence of Hsp70 (41). These differences could explain why only Ydj1 synergizes with Ssa1 during TA capture, whereas Sis1 did not. Importantly, Sis1 can replace Ydj1 to support efficient TA insertion into the ER *in vivo* (Figs. 1 and 6) despite its inability to assist Ssa1 in TA trapping. This strongly suggests that the roles of JDPs in facilitating the ER-targeting of TAs cannot be solely attributed to enhanced TA trapping by Hsp70.

Unexpectedly, an activity shared between Ydj1 and Sis1 during their participation in the GET pathway is to improve the transfer of Ssa1-bound TA onto the downstream cochaperone Sgt2. In contrast to the initial TA trapping event that requires the client binding domain of Ydj1, the Ydj1 J-domain is necessary and sufficient to enhance this transfer. Moreover, Sis1 enhanced this transfer as efficiently as Ydj1 despite its inability to synergize with Ssa1 during initial TA capture. These observations exclude the possibility that this enhancement was because of better capture and solubilization of TA by the JDP/Hsp70 pair and suggest instead that JDP-induced changes in the conformation of the Ssa1·TA complex are responsible for the improvement in client transfer. The loss of this enhancement by the H34Q mutation in both JDPs or the T201A mutation in Ssa1 further indicates that the JDP-induced ATPase activation of Ssa1 is required for the enhanced TA transfer competence. While this observation is counter-intuitive, as Hsp70 binds client proteins more tightly in the ADP-state because of lid closing in the SBD (52, 53, 54), there is growing evidence that ADP-bound Hsp70 does not fully close the lid when it is loaded with protein substrates (5, 6, 55). We propose that the Ydj1 and Sis1 J-domains regulate the conformation and nucleotide state of Ssa1, inducing it to bind TAs in a conformation that is more conducive to subsequent loading on Sgt2. These results extend the recent works showing that DnaJ/K can release client proteins in a more folding-competent conformation (15) and demonstrate that JDPs play an essential role in this client conformational regulation during the biogenesis of an essential class of integral membrane proteins. The enhanced folding competence of client proteins as a result of cooperation between JDP and Hsp70 could be envisioned in the biogenesis of a broad array of Hsp70 clients (32).

**Figure 7:**
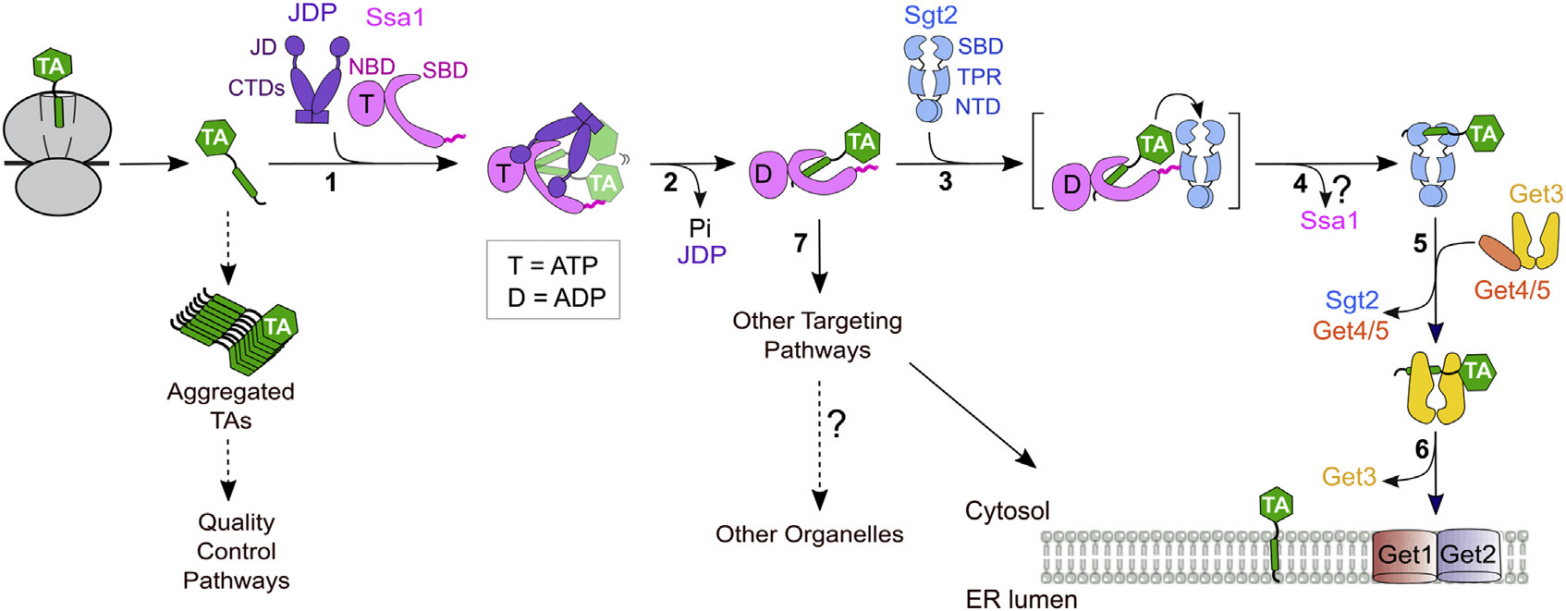
**Revised model of TA targeting by the GET pathway in yeast.** Step 1 to 2, TAs released from the ribosome are captured by Ssa1 and protected from aggregation in the cytosol (*dashed arrows*). The efficiency of this step can be further enhanced by Ydj1 but not Sis1. The J-domain of Ydj1 or Sis1 stimulates ATP hydrolysis on Ssa1 and drives it to bind TA in an altered conformation. Step 3, the TA transfer complex is assembled *via* association of Ssa1 with the Sgt2 TPR domain. The Ydj1- or Sis1-induced conformational changes in Ssa1 also enhance the transfer competence of the TA. Step 4, TA is transferred from Ssa1 to Sgt2. The question mark indicates that it is unclear whether Ssa1 dissociates from Sgt2 after TA transfer. Step 5, TAs undergo a second transfer from Sgt2 to Get3 assisted by the Get4/5 complex. Step 6, Get3 delivers TAs to the Get1/2 receptors for insertion into the ER membrane. Step 7, TAs bound to JDP·Ssa1 could enter alternative targeting pathways to be targeted to the ER or to other organelles. JDP, J-domain protein; TA, tail-anchored protein.

The roles of Ydj1/Sis1 in generating a more targeting-competent conformation of the Ssa1·TA complex may not be limited to the GET pathway. Previous work suggested the presence of multiple redundant pathways in yeast that together form a robust network for the targeting of TAs and other membrane proteins to the ER (34, 36, 56). Curiously, deletion of Sgt2 has a much weaker phenotype than the deletion of downstream genes including Get3 and Get1/2 (56, 57, 58), which was suggested to reflect the increased commitment of TA substrates to the GET pathway as they engage factors downstream of Sgt2. In support of this notion, Sbh1 forms cytosolic aggregates in *Δget2* cells but is efficiently inserted into the ER membrane in *Δsgt2Δget2* cells (56), indicating that TAs can be readily re-routed to alternative targeting pathways in the absence of Sgt2. Another puzzling observation is that transient inactivation of Ssa1 or depletion of Ydj1/Sis1 caused significantly larger defects in TA insertion than the deletion of Sgt2 (this work and (31)). This may be explained by the observation in this work that Ssa1 together with Ydj1/Sis1 are also required for the efficient *in vivo* targeting of GET-independent TAs as well as SND substrates. Collectively, these results suggest a model in which the cytosolic JDP/Hsp70 forms a chaperone hub upstream of multiple, redundant membrane protein insertion pathways (Fig. 7, step 7). The ability of Ydj1/Sis1 to generate a more targeting-competent conformation of TA on Ssa1 could contribute to enhanced TA insertion *via* the alternative pathways, and possibly also to the insertion of mitochondrial TAs as recently described (25).

We propose a new working model for the Hsp70/40-mediated biogenesis of TAs in the GET pathway. The low intrinsic ATPase activity of Ssa1 (59) and the 10-fold excess of ATP over ADP *in vivo* maintain most of the free Hsp70s in the ATP-bound state. In this state, Ssa1 can rapidly capture newly synthesized TAs and protect them from irreversible aggregation in the aqueous cytosolic environment (Fig. 7, step 1 and *dashed arrows*). A class A JDP, Ydj1, can assist Hsp70 in this initial capture, although this activity is not shared by the class B JDP, Sis1, and does not appear to be required. Both Ydj1 and Sis1 use their J-domains to activate ATP hydrolysis in Ssa1 and alter the conformation of the Ssa1·TA complex (Fig. 7, step 1–2). The Ssa1·TA complex recruits the cochaperone Sgt2 *via* interaction of the Ssa1 C-terminal motif with the Sgt2 TPR domain and transfers the bound TA to Sgt2 (Fig. 7, step 2–3). The JDP-induced changes in the conformation and nucleotide state of Ssa1 generate a more active transfer complex in which the TA is loaded onto Sgt2 with higher efficiency (Fig. 7, transfer complex in bracket). With the help of Get4/5, Sgt2 further hands off the TA substrate to the targeting factor Get3, thus committing the TA for delivery to the ER (Fig. 7, step 4–6). The JDP·Ssa1·TA complex could also be directed into alternative pathways if the TA fails to engage with Sgt2 (Fig. 7, step 7).

## Experimental procedures

### Strains

YTJ100 (YMK120a ydj1::tetO7-Ubiquitin-Leu-YDJ1(NatMX)), YTJ102 (YMK120a sis1::tetO7-Ubiquitin-Leu-SIS1(NatMX)), and YTJ138 (YMK120a sis1::tetO7-Ubiquitin-Leu-SIS1 (NatMX); ydj1::tetO7-Ubiquitin-Leu-YDJ1 (KanMX)) were kindly provided by Doron Rapaport (25). BY4741 (*WT*) and *Δget3* (BY4741 *YDL100C*::*KanMX*) strains were purchased from American Type Culture Collection. *SSA1* (*MATα leu2-3112 his3-11 ura3-52 trp1*Δ*1 lys2 ssa2*::*LEU2 ssa3::TRP1 ssa4*::*LYS2*) and *ssa1*^*ts*^ (*MATα leu2-3112 his3-11 ura3-52 trp1Δ1 lys2 ssa1-45*:*URA3 ssa2*::*LEU2 ssa3*::*TRP1 ssa4*::*LYS2*) strains were kindly provided by Dr Elizabeth A. Craig (37).

### Protein expression and purification

Strep-SUMO-Bos1 and His_6_-Sgt2 were expressed and purified as described previously (31, 35).

To express Strep-SUMO-Bos1^Bpa^, the coding sequence for the eighth amino acid residue (Ile) in the Bos1-TMD (LVFWIALILLIIGIYYVL) was replaced by an amber codon (TAG) using the expression plasmid for Strep-SUMO-Bos1 (31) as a template and QuikChange mutagenesis (Agilent Technologies, Inc.). Expression plasmids for Strep-SUMO-Bos1^Amb^ and tRNA_CUA_^Opt^ synthetase (43) were co-transformed into BL21 Star (DE3) cells (Thermo Fisher Scientific Inc.). The expression of tRNA_CUA_^Opt^ synthetase was induced at OD_600_ of 0.4 using 0.2% arabinose and then 1 mM Bpa was supplemented in the media. At OD_600_ of 0.6, expression of Strep-SUMO-Bos1^Amb^ was induced with 0.1 mM isopropyl β-D-1-thiogalactopyranoside for 100 min at 37 °C. Bpa incorporation into Bos1 was confirmed by SDS-PAGE analysis. The purification of Strep-SUMO-Bos1^Bpa^ was performed as described previously, and then, the purified protein was stored in buffer A (50 mM Hepes [pH 7.5], 150 mM NaCl, 10% glycerol, 0.05% LDAO) (31).

His_6_-SUMO-Ssa1, His_6_-SUMO-Ssa1-T201A, His_6_-SUMO-Ydj1, His_6_-SUMO-Ydj1-H34Q, His_6_-SUMO-Ydj1-JD-GF(1–102aa), His_6_-SUMO-Sis1, His_6_-SUMO-Sis1-H34Q, His_6_-SUMO-Sis1-K199N/K202N/K214N (3KN), and His_6_-SUMO-Sis1-JD-GF(1–121aa) were expressed and purified as previously described (31) with minor modifications. After protein expression, cells were resuspended in buffer B (20 mM Tris [pH 8.0], 500 mM NaCl, 10% glycerol, 2 mM β-ME, 15 mM imidazole) supplemented with protease inhibitor cocktail. Clarified lysate was incubated with Ni Sepharose High Performance resin (GE Healthcare). The column was washed with buffer B and eluted with buffer B containing 300 mM imidazole. To obtain tagless Ssa1, Ydj1, and their mutants, purified His_6_-SUMO fusion proteins were digested with SUMO protease overnight at 4 °C, and the proteins were further purified using either MonoQ 5/50GL or MonoS 5/50GL (GE Healthcare).

### Turbidity assay

90 μM Strep-SUMO-Bos1 stored in buffer A was rapidly (within 15 s) diluted to a final concentration of 1.5 μM in assay buffer (20 mM K-Hepes [pH 7.5], 150 mM KOAc, 5 mM Mg(OAc)_2_, 2 mM β-ME, 2 mM ATP) containing indicated concentrations of chaperones (Ssa1 or/and Ydj1). The optical density at 360 nm was measured in real time using Spectrophotometer DU 640 (Beckman Coulter). The observed solubility of Bos1 (S_obsd_) was calculated from the % change of optimal readings at 5 min between Bos1 alone and chaperone-containing samples. The data were plotted as a function of Ssa1 concentration and fit to Equation 1,

(1) $${\text{S}}_{obsd}={\text{S}}_{Max}\times \frac{\left[Ssa1\right]}{K_{soluble}+\left[Ssa1\right]}$$

where S_Max_ is the % soluble TA at saturating Ssa1 concentrations, and *K*_soluble_ is the apparent TA binding constant.

### Sedimentation analysis of recombinant Bos1

90 μM Strep-SUMO-Bos1 stored in buffer A was diluted to a final concentration of 3 μM in assay buffer containing indicated concentrations of chaperones and incubated at RT for 5 min. The reactions were ultracentrifuged at 100,000 rpm for 30 min using TLA 100 rotor (Beckman Coulter). Total input (T), soluble (S), and pellet (P) fractions were resolved by SDS–PAGE and detected by Western blot using the following antibodies: Bos1, anti-Strep; Sgt2 or Ydj1, anti-His; Ssa1, anti-Ssa (a gift from Elizabeth A. Craig). Western blots were imaged using Odyssey Imager (LI-COR Inc.) and quantified using Quantity One software (Bio-Rad). The concentration of soluble TA (*S*_*obsd*_) was calculated as [Total Bos1 concentration∗I_S_/I_T_], where I_S_ and I_T_ denote the intensities of the bands of S and T fractions, respectively. The Ssa1 concentration dependence of *S*_*obsd*_ values was fit to Equation 2,

(2) $$S_{obsd}={\text{S}}_{Max}\times \left\{\frac{\left[\text{TA}\right]+\left[\text{Ssa}1\right]+K_{Soluble}-\sqrt{{\left(\left[\text{TA}\right]+\left[\text{Ssa}1\right]+K_{Soluble}\right)}^{2}-4\left[TA\right]\left[Ssa1\right]}}{2\left[TA\right]}\right\}$$

in which S_Max_ is the concentration of Bos1 at saturating Ssa1 concentrations, and *K*_Soluble_ is the apparent binding constant of Ssa1 or Ssa1·Ydj1 to Bos1.

### TA transfer reaction from Ssa1 to Sgt2

80 μM Strep-SUMO-Bos1^Bpa^ stored in buffer A was diluted to a final concentration of 0.1 μM in assay buffer containing 3 μM Ssa1 or/and 3 μM Ydj1 and incubated for 1 min at room temperature. His_6_-Sgt2 was added to a final concentration of 0.3 μM in the reaction (100 μl), and reactions were further incubated at room temperature. At indicated time points, 10 μl aliquots were removed from the reaction and quenched by flash freezing in liquid nitrogen. 0 s samples were taken right before Sgt2 was added in the reactions. Frozen aliquots were subsequently crosslinked on dry ice ∼4 cm away from a UVP B-100AP lamp (UVP LLC) for 90 min. Crosslinked bands were resolved on SDS–PAGE and analyzed by Western blot. Crosslinking efficiency was calculated as [I_Bos1xSgt2_/(I_Bos1_ + I_Bos1xSgt2_ +I_Bos1xYdj1_)]∗100, where I denotes the intensity of the band of interest.

### Single turn-over ATPase assay

3 μM Ssa1 with or without 3 μM Ydj1 variants in reaction buffer (20 mM K-Hepes [pH 7.5], 150 mM KOAc, 5 mM Mg(OAc)_2_, 5 mM β-ME) was mixed with 2.4 μM ATP containing γ^32^P-ATP and incubated at room temperature. At indicated time points, 2 μl aliquots were removed from the reaction and quenched in 4 μl TLC solution (500 mM LiCl, 1 M Formic acid). γ^32^P-ATP and γ^32^P-Pi were separated by thin layer chromatography using PEI cellulose (Vendor) and quantified by phosphorimaging using a Typhoon imager (GE Healthcare). Time courses of the reaction were fit to Equation 3,

(3) $${\left[ATP\right]}_{t}=a\times e^{-k_{obsd}t}$$

in which *a* is the fraction of ATP before initiation of the reaction, and *k*_*obsd*_ is the observed rate constant for Ssa1 ATP hydrolysis.

### Pulse-chase experiments

Plasmids (pRS313-BirA-Bos1, pRS313-Bos1-BirA, pRS313-DHC-αF, pRS313-BirA-6AG, and pRS313-Scs2-GFP) were transformed into indicated yeast cells. Colonies were grown in selective media (SD-His) until OD_600_ reached 0.2. Cells were supplemented with 2 μg/ml doxycycline (Sigma-Aldrich) and cultured for an additional 4 h at 30 °C. Cells were then washed with water and resuspended in SD-His-Met media at 8 OD_600_ units per ml. After growing at 30 °C for 30 min, cells were pulse-labeled with 200 μCi/ml EasyTagTM EXPRESS35S Protein Labeling Mix (PerkinElmer) for 2 min and chased with 10 mM cold methionine. Cells (200 μl) were removed from the culture and flash-frozen in liquid nitrogen at indicated times during the chase.

Pulse-chase experiments with BirA-6AG and Scs2-GFP in *SSA1* and *ssa1*^*t*^ cells were performed as previously described (31).

For complementation assays, Ydj1 or Sis1 variants (pESC-Trp-Myc-Ydj1, pESC-Trp-Myc-Ydj1-H34Q, pESC-Trp-Myc-Sis1, pESC-Trp-Myc-Sis1-H34Q, and pESC-Trp-Myc-Sis1-3KN) were co-transformed with pRS313-BirA-Bos1 into YTJ138 (YMK120a sis1::tetO7-Ubiquitin-Leu-SIS1 (NatMX); ydj1::tetO7-Ubiquitin-Leu-YDJ1 (KanMX)) cells. Colonies were grown in SD-His-Trp media overnight, and cells were washed and resuspended in selective media containing 2% Raffinose and 0.05% Glucose. After adjusting OD_600_ to ∼0.2, cells were cultured for 4 h at 30 °C, and 2% galactose and 2 μg/ml doxycycline were supplemented. Cells were further cultured for 4 h at 30 °C, and pulse-chase experiments were performed as described above.

The frozen cell samples were lysed and immunoprecipitated using anti-HA magnetic beads as previously described (31). Immunoprecipitated samples were resolved by SDS–PAGE and quantified by autoradiography. Translocation efficiency was calculated as [I_glycosylated_/(I_non-glycosylated_ + I_glycosylated_)]∗100, where I denotes the intensity of the band of interest.

### Quantification and statistical analysis

Statistical details of each experiment can be found in the figure legends. For both Western blot and radiographic images, Quantity One 1-D Analysis Software (Bio-Rad) was used to quantify the intensities of bands. The value of n is specified in each figure legend, as well as the *p* value when statistical test was performed.

## Data availability

All data described are contained in the manuscript.

## Supporting information

This article contains supporting information.

## Conflict of interest

The authors declare that they have no conflicts of interest with the contents of this article.
