# Supplementary material for: J-domain proteins promote client relay from Hsp70 during tail-anchored membrane protein targeting
Source: J Biol Chem. 2021 Mar 17;296:100546. doi: 10.1016/j.jbc.2021.100546 (PMC8054193; doi:10.1016/j.jbc.2021.100546)
Supplement: Supplementary file 1 — Figures S1 to S8 [file mmc1.pdf]

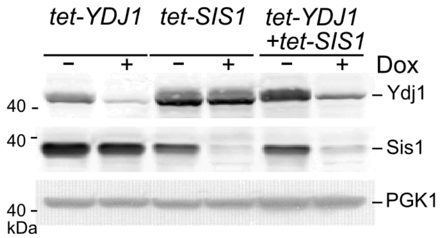

Figure S1. JDP expression was substantially reduced upon addition of doxycycline.

The indicated yeast cells were incubated without and with 2 μg/mL Doxycycline (Dox) for 4 hr at 30 °C, and the lysates were used for western blot analyses. PGK1 serves as a loading control.

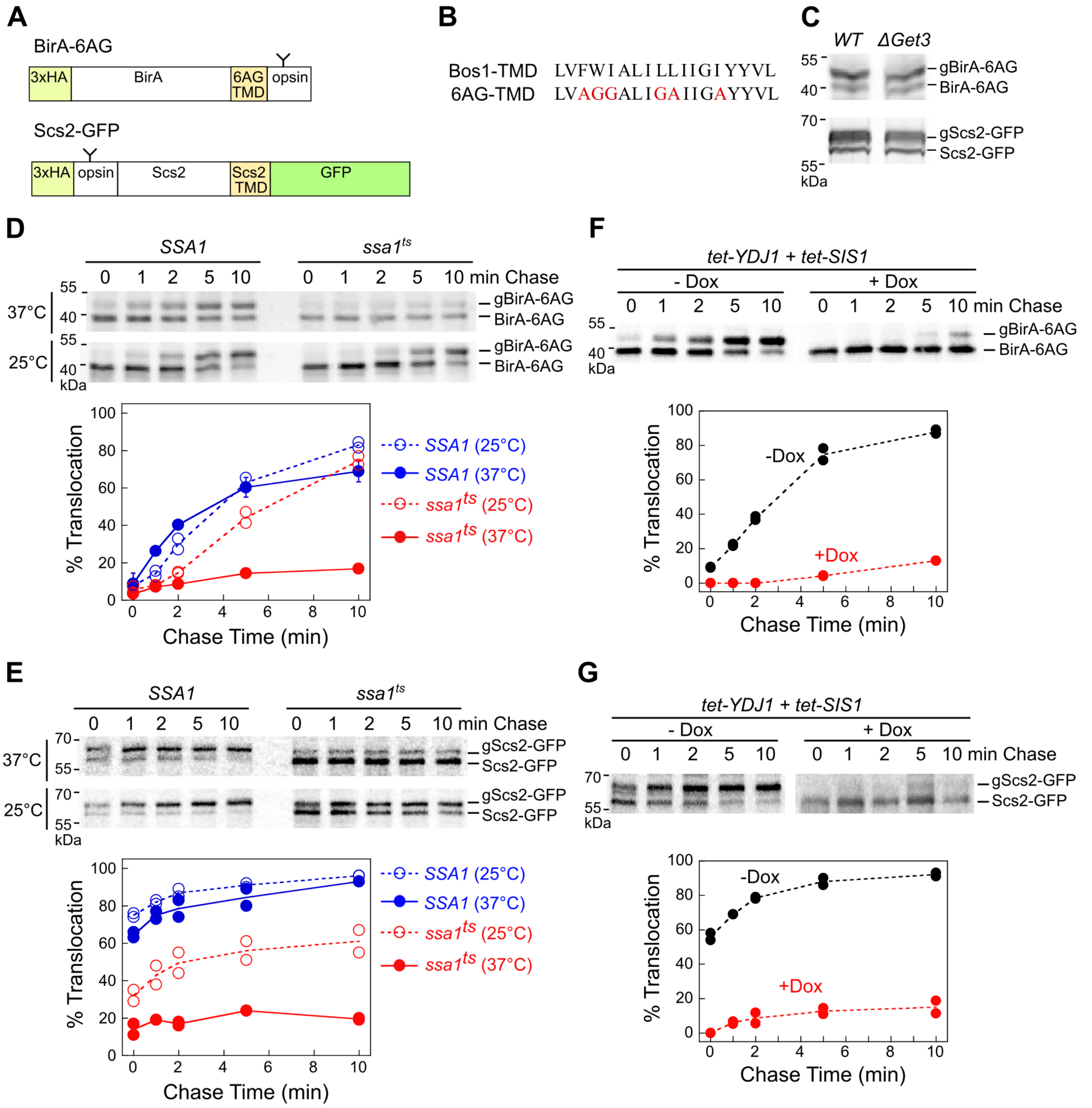

Figure S2. The Hsp70/JDP system is required for the efficient ER targeting of GET-independent TAs and SND substrates.

(A) Scheme of the model substrates used to assay *in vivo* targeting via the GET-independent (6AG) and SND (Scs2-GFP) pathways. (B) The TMD sequences of BirA-Bos1 and BirA-6AG. (C) Steady-state translocation levels of BirA-6AG and Scs2-GFP in *WT* and Δ*get3* cells. Glycosylated and non-glycosylated proteins were resolved by SDS–PAGE and detected by anti-HA antibody. (D) and (E) Top, representative autoradiograms for pulse-chase analysis of the translocation of metabolically labeled BirA-6AG and Scs2-GFP in *SSA1* (blue) and *ssa1^{ts}* (red) cells at 25 °C (dashed lines and open circles) and 37 °C (solid lines and

closed circles). Both SSA1 and *ssa1*^{ts} cells are functional at permissive temperature (25 °C). Upon switching to nonpermissive temperature (37 °C) for 5 min, Ssa1 was inactivated in *ssa1*^{ts} cells, whereas SSA1 cells are unaffected (36). Bottom, quantifications of the data in (D), (E), and their replicates. (F) and (G) Top, representative autoradiograms for pulse-chase analysis of the translocation of metabolically labeled BirA-6AG (F) and Scs2-GFP (G) in tet-YDJ1/tet-SIS1 cells in the absence (-) and presence (+) of Doxycycline (Dox). Bottom, quantification of the data in (F) and (G) and their replicates. In (D) – (G), values from two biological replicates are shown as circles. The values for *SSA1* and *ssa1*^{ts} at 37 °C in (D) represent mean ± SD, with n = 3 (biological replicates).

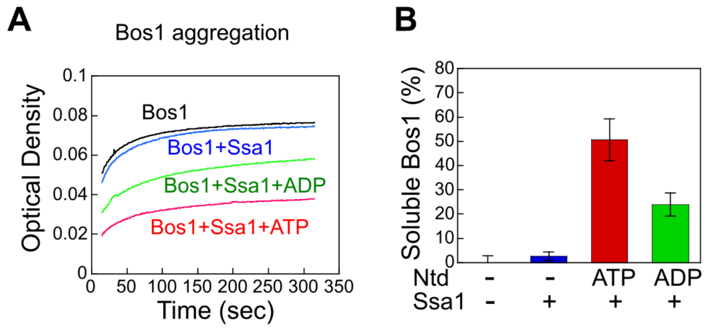

Figure S3. Nucleotide binding on Ssa1 is required for its ability to capture TAs in the soluble form.

(A) Time courses of Bos1 aggregation measured by the turbidity assay in the absence or presence of Ssa1 and in the presence of the indicated nucleotides. (B) Quantification of soluble Bos1 from the data in (A) and their replicates. All values in (B) are reported as mean ± SD, with n=3.

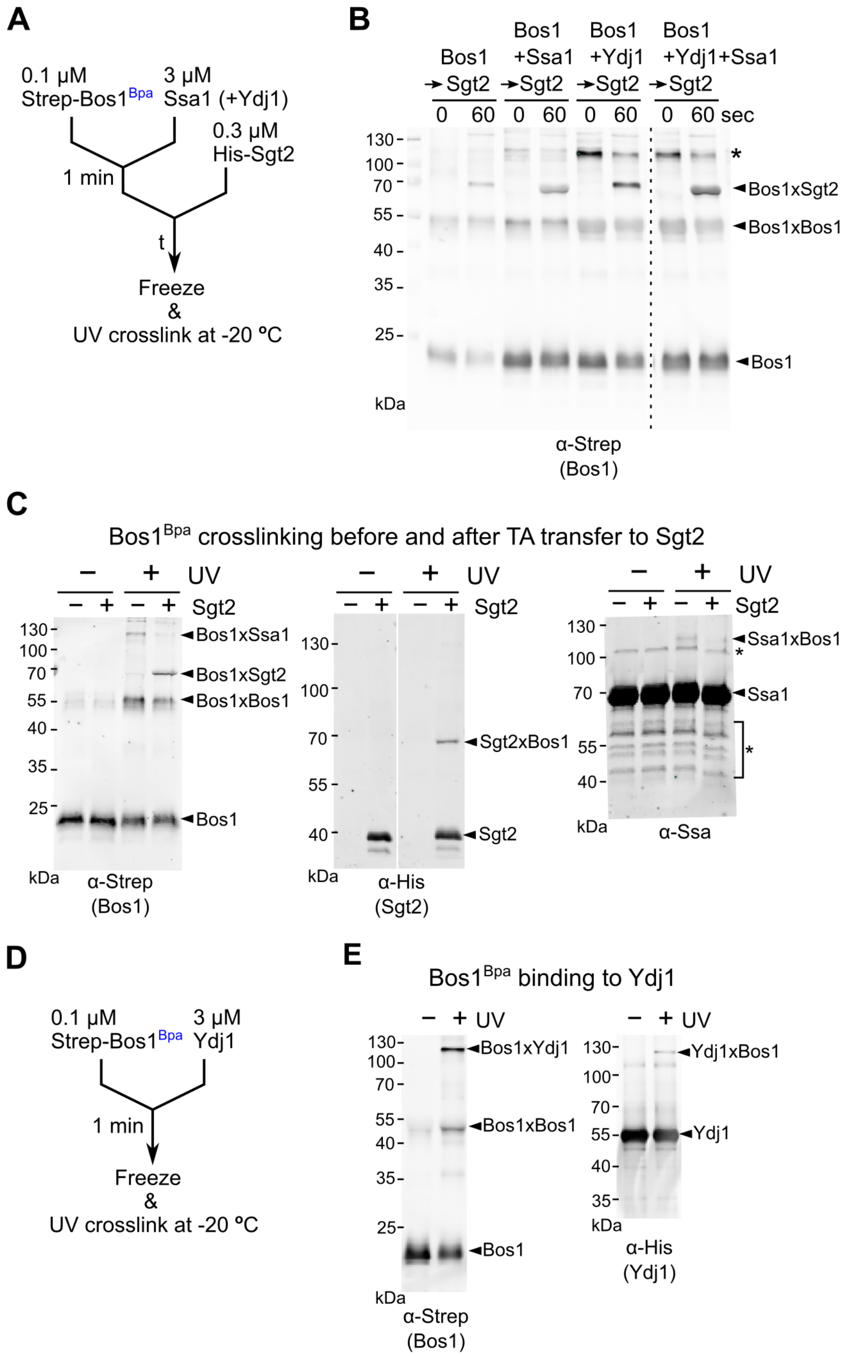

Figure S4. Western blot analyses of Bos1^{Bpa} and its crosslinks to different chaperones in the GET pathway.

(A) Scheme of the assay used in (B) and (C) to measure Bos1^{Bpa} transfer from Ssa1 to Sgt2. (B) Anti-Strep (Bos1) western blot showing Bos1^{Bpa} and its crosslink to different chaperones. The dashed line indicate that the images are from the same gel. In lanes 1-2, Bos1^{Bpa} was directly added to Sgt2. In lanes 3-8, Bos1^{Bpa} was pre-incubated with Ssa1 (lanes 3-4), Ydj1 (lanes 5-6), or both (lanes 7-8*)* followed by addition of Sgt2.

Samples were flash frozen and UV-crosslinked before (t = 0) and 60 sec after Sgt2 addition. * denotes the Bos1xSsa1 or Bos1xYdj1 crosslink that both run at ~125 kDa. (C) Western blot images showing Bos1^{Bpa} and its crosslinks to Ssa1 and Sgt2 before and after the Ssa1-to-Sgt2 TA transfer, carried out as in Fig. S4A. Samples before (–Sgt2) and 5 min after (+Sgt2) the initiation of transfer were UV-crosslinked where indicated. Bos1, Sgt2, and Ssa1 were detected using anti-Strep (left), anti-His (middle), anti-Ssa (right) antibodies, respectively. The asterisk denotes non-specific bands detected by the anti-Ssa antibody. The crosslinked Bos1 dimer (Bos1xBos1) was not interpreted in this study. (D) Scheme of the assay used in (E) to measure Bos1^{Bpa} binding to Ydj1. 0.1 μM Bos1Bpa was incubated with 3 μM His-Ydj1 for 1 min, followed by UV crosslinking. (E) Western blot images showing Bos1^{Bpa} and its crosslink to Ydj1. Bos1 and Ydj1 were detected using anti-Strep (Bos1; left) and anti-His (Ydj1; right) antibodies.

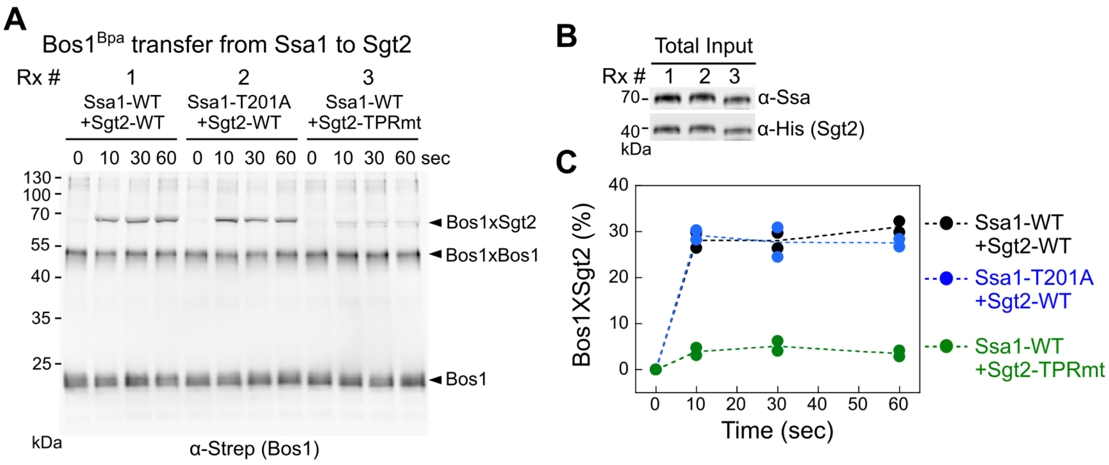

Figure S5. The transfer of Bos1 from Ssa1 to Sgt2 is dependent on the interaction of Ssa1 with the Sgt2 TPR domain.

(A) A representative western blot analysis of the time courses of Ssa1-to-Sgt2 Bos1^{Bpa} transfer with the indicated Ssa1 and Sgt2 variants. The assay was performed as described in Fig. S4A. (B) Western blot analyses of the amounts of Ssa1 and Sgt2 for the reactions in (A). (C) Quantification of the amount of the Bos1-Sgt2 crosslink (Bos1xSgt2) from the data in (A) and their replicates. Values from two independent experiment are shown as circles.

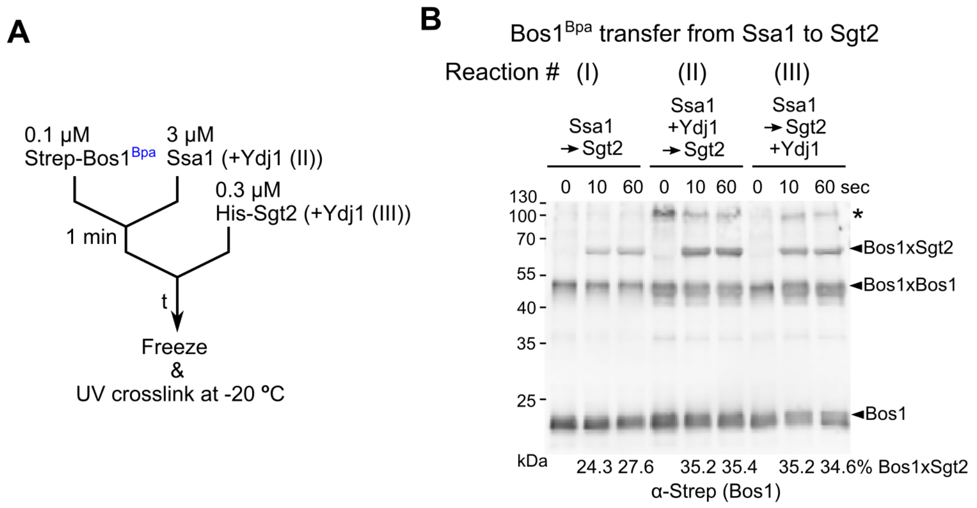

Figure S6. Ydj1 enhances TA transfer from Ssa1 to Sgt2 when added after the initial TA solubilization.

(A) Scheme of the assay used in (B) to measure Bos1^{Bpa} transfer from Ssa1 to Sgt2. (B) Western blot image of the Ssa1-to-Sgt2 TA transfer when Bos1^{Bpa} was pre-incubated with Ssa1 (Reaction I (lanes 1-3)) or Ssa1 supplemented with Ydj1 (Reaction II (lanes 4-6)), or when Ydj1 was added together with Sgt2 after pre-incubation of Bos1^{Bpa} with Ssa1 (Reaction III (lanes 7-9)).

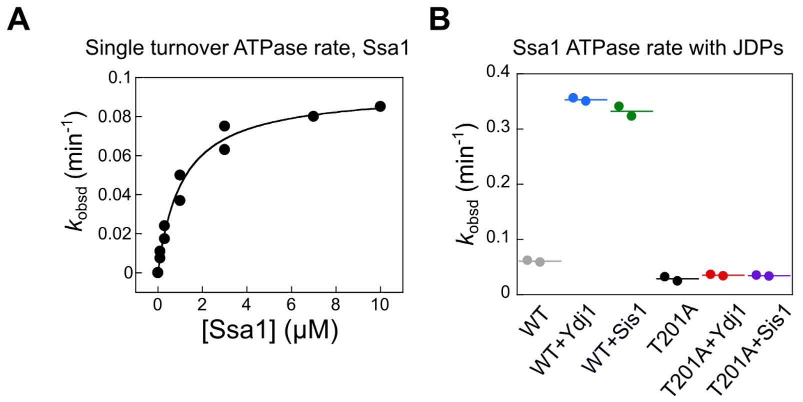

Figure S7. The Ssa1(T201A) mutation abolished the JDP-induced ATPase activation of Ssa1.
(A) Single turnover ATPase rate constant of Ssa1 was determined as a function of Ssa1 concentration, as described in the Methods. The data were fit to the Michaelis-Menten equation, which gave a k_{cat} value of 0.091 min^{-1} and a K_M value of 1.1 μM. (B) Single turnover ATPase rate of 3 μM wildtype (WT) Ssa1 or mutant Ssa1(T201A) were determined with and without 3 μM Ydj1 or Sis1 present. Values from two independent experiments are shown in circles, with the mean values represented by the lines.

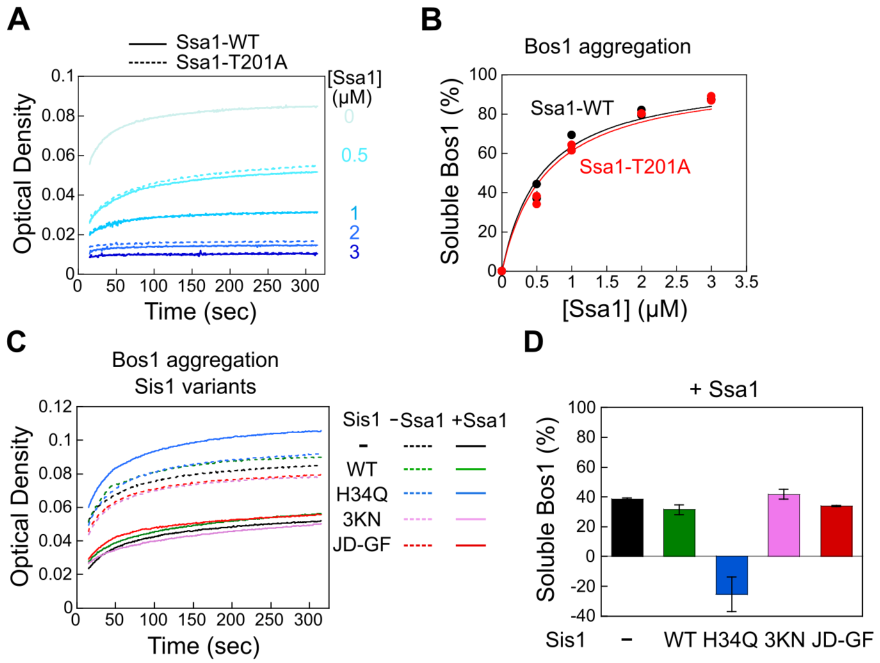

Figure S8. Ssa1(T201A) does not affect the chaperone activity of Ssa1 towards TAs in the absence of JDPs, and none of the Sis1 variants assisted Ssa1 in TA capture.

(A) Time courses of Bos1 aggregation in the presence of Ssa1-WT (solid lines) or Ssa1-T201A (dotted lines). (B) Quantification of the data in (A) and their replicates. The lines are fits of the data to Equation 1 and yielded apparent TA binding constants (K_{Soluble} values) of 0.58 ± 0.05 and 0.63 ± 0.08 μM for Ssa1-WT and Ssa1-T201A, respectively. The values from two independent experiments are shown as black (Ssa1-WT) and red (Ssa1-T201A) circles. (C) Time courses of Bos1 aggregation in the presence of 0.5 μM of indicated Sis1 variants with (solid lines) and without (dotted lines) 0.5 μM Ssa1. (D) Quantification of the data in (C) and their replicates. All values in (D) are reported as mean ± SD, with n = 3. Error bars are shown but may not be visible in some cases.
